# Cardiotrophin-1 is inversely associated with obesity in non-diabetic individuals

**DOI:** 10.1038/srep17438

**Published:** 2015-12-01

**Authors:** Hao-Chang Hung, Feng-Hwa Lu, Hung-Tsung Wu, Horng-Yih Ou, Yi-Ching Yang, Jin-Shang Wu, Chih-Jen Chang

**Affiliations:** 1 Division of Endocrinology and Metabolism, Department of Internal Medicine, National Cheng Kung University Hospital, Tainan, Taiwan; 2 The Institute of Basic Medical Sciences of National Cheng Kung University, Tainan, Taiwan; 3 Department of Family Medicine, National Cheng Kung University Hospital, Tainan, Taiwan; 4 College of Medicine, National Cheng Kung University, Tainan, Taiwan

## Abstract

Cardiotrophin-1 is known to be a key regulator of energy homeostasis, as well as glucose and lipid metabolism *in vivo*. However, there are inconsistent results of the association between cardiotrophin-1 and obesity in humans, possibly confounded by hyperglycemia. Therefore, the aim of this study was to investigate the relationships among cardiotrophin-1 levels, overweight and obese individuals without diabetes in a Chinese population. The median (inter-quarter range) serum cardiotrophin-1 levels were 447.9 (230.9, 913.9), 350.6 (201.1, 666.5), and 288.1 (162.3, 572.4) pg/ml in non-diabetic subjects who were of normal weight (n = 522), overweight (n = 203), and obese (n = 93), respectively (trend test *p* < 0.001). Subjects who were overweight and obese had significantly lower cardiotrophin-1 levels than those with normal weight. The multivariate linear regression analyses showed that overweight (beta = −338.718, 95% CI = −552.786 ~ −124.651, p < 0.01), obese (beta = −530.275, 95% CI = −832.967 ~ −227.583, p < 0.01), and smoking (beta = −377.375, 95% CI = −654.353 ~ −100.397, p < 0.01) were negatively related to cardiotrophin-1 after adjusting for age, gender, HOMA-IR, hypertension, total cholesterol, HDL, triglyceride, eGFR, ALT, and alcohol drinking. The results of this study provided epidemiological evidence that non-diabetic subjects who were overweight or obesity had significantly lower cardiotrophin-1 concentrations than those with normal weight, and both obesity and being overweight were inversely associated with cardiotrophin-1 levels.

The interleukin-6 (IL-6) family of cytokines, including cardiotrophin-1, IL-6, IL-11, leukemia inhibitory factor (LIF), oncostatin M (OSM), and ciliary neurotrophic factor (CNTF), regulates a variety of biological process, such as immune response, inflammation, hematopoiesis, cardiovascular action, and neuronal survival^1^. These cytokines share glycoprotein 130 (gp130) as a common signal transducer within their receptor complex, and the gp130 ligands may act as potential therapeutic targets in obesity^2^. Of these, cardiotrophin-1 was identified by expression cloning from a house embryonic stem cell-based model of cardiogenesis that potently induces cardiac myocyte hypertrophy *in vitro*^3^. It is associated with both left ventricular hypertrophy and left ventricular dysfunction in patients with hypertensive heart disease, and cardiotrophin-1 might be a potential marker of the development, progression, and regression of this condition^4^.

Recently, growing interest has been focused on the role of cardiotrophin-1 in the metabolism. Cardiotrophin-1 is known to be a key regulator of energy homeostasis, as well as the glucose and lipid metabolism in adipose tissue, muscle^5^, and in liver^6^. Mice lacking cardiotrophin-1 developed metabolic abnormalities, characterized by obesity, hyperglycemia, hyperinsulinemia, and hypercholesterolemia^5^. In addition, chronic cardiotrophin-1 treatment reduced body weight, improved insulin resistance^5^, and resolved hepatic steatosis^6^ in *ob/ob* and high-fed-diet obese mice, suggesting that cardiotrophin-1 is a promising therapy for obesity, diabetes, and non-alcoholic fatty liver disease.

In contrast with the experimental research on the role of cardiotrophin-1 in the metabolism, there are inconsistent results on the relationship between cardiotrophin-1 and obesity in human studies. In a cohort of 81 patients (median age 68 years) with a high prevalence of diabetes (30.9%), hypertension (81.5%), and metabolic syndrome (44.4%) who were undergoing coronary angiography, obese subjects showed significantly lower cardiotrophin-1 levels than non-obese ones, and cardiotrophin-1 levels were inversely correlated with BMI^7^. In 44 overweight/obese children participating in a weight loss program, individuals with higher BMI had lower cardiotrophin-1 values^8^. In a study of 72 adolescents, overweight and normal weight subjects did not differ in cardiotrophin-1 levels, and BMI did not correlate with cardiotrophin-1^9^. In a population of 137 apparently healthy subjects, increased cardiotrophin-1 levels were observed in obese patients compared with normal weight subjects^10^. Our previous study that enrolled 265 subjects revealed that obese, diabetic individuals had significantly lower cardiotrophin-1 levels than non-obese diabetic subjects, and obesity was negatively related to cardiotrophin-1 levels, while impaired glucose tolerance and newly diagnosed diabetes were positively related to it^11^. These discrepant results may be due to the relatively small sample sizes and lack of adjustment for possible confounding factors of cardiotrophin-1 in the previous studies, especially hyperglycemia, which has been shown to increase the protein expression of cardiotrophin-1 in adipose tissue^10^. However, there are currently no studies on cardiotrophin-1 in obese subjects without hyperglycemia. The aim of this study is thus to investigate the relationships among cardiotrophin-1 levels, overweight and obese individuals without diabetes in a Chinese population.

## Materials/Subjects and Methods

The subjects were recruited for the population-based study for chronic diseases conducted in Tainan, and the details of its results have been previously described^12^. Briefly, a three-stage sampling scheme was used to generate a stratified systemic cluster sample of households throughout the city. All of the subjects who were aged 20 years or more according to the government population register in 1995 were included in the study. Of the 2,416 eligible people, 1,638 subjects, representing a response rate of 67.8%, finished a health screening examination. Informed consent was obtained from all participants, and the study was approved by the Institutional Review Board of the National Cheng Kung University Hospital. The methods were carried out in accordance with the approved guidelines. Subjects with the following conditions or diseases were excluded: 1) a history of heart failure, myocardial infarction, or ischemic heart disease; 2) symptoms of heart failure or ischemic heart disease; 3) left ventricular hypertrophy diagnosed by electrocardiogram; 4) diabetes mellitus; 5) any acute or chronic inflammatory disease, as determined by a leukocyte count of more than 10,000/mm^3^ or clinical signs of infection; 6) any other major diseases, including generalized inflammation or advanced malignant diseases contraindicating this study; 7) alcohol consumption ≥20 g/day in the last year; and 8) serum aspartate aminotransferase or alanine aminotransferase levels greater than two times the upper normal limit.

With the help of specially trained assistants, all subjects were interviewed according to a structured questionnaire. The questionnaire included demographic information, past medical history, medication history, current smoking habit, and current drinking habit over the past year. All the subjects received a physical examination by physicians. Height and weight were measured in light clothing without shoes. BMI (in kg/m^2^) was calculated as weight (in kilograms) divided by height (in meters) squared. Being overweight or obese were defined according to the recommendations of the Health Promotion Administration in Taiwan, obesity as a BMI ≥ 27, overweight as a 27 > BMI ≥ 24, and normal weight as a 24 > BMI ≥ 18.5 kg/m^2^. Two readings of systolic and diastolic blood pressure were measured in the sitting position with a DINAMAP vital signs monitor (Model 1846SX, Critikon Inc., Irvine, CA) after a 15-min rest period. Subjects with a systolic blood pressure (SBP) ≥ 140 mmHg, or diastolic blood pressure (DBP) ≥ 90 mmHg, or a history of hypertension were defined as having hypertension. All patients were examined using 12-lead electrocardiography (ECG). Smoking habit was classified into smokers (defined as at least one pack/month over the past year) and non-smokers. Alcohol drinking was classified into drinkers (defined by at least one drink per week over the past year) and non-drinkers.

The laboratory tests included routine biochemistry, hemogram, fasting plasma glucose, total cholesterol, triglyceride, HDL, insulin, and cardiotrophin-1 after an overnight fast of at least 10 hours. The subjects without a history of diabetes also received a 75-g OGTT. Blood glucose was measured by a hexokinase method (Roche Diagnostic GmbH, Mannheim, Germany). Diabetes mellitus was defined by past history of diabetes, or use of anti-diabetic medications, or FPG ≥7.0 mmol/l, or 2-h postload glucose ≥11.1 mmol/l. Serum insulin (Mercodia AB, Uppsala, Sweden) was measured by ELISA. Insulin resistance was defined by the homeostasis model assessment-insulin resistance (HOMA-IR) index as [fasting insulin (μU/ml) × fasting plasma glucose (mM)]/22.5. The measurement of serum cardiotrophin-1 was carried out using human cardiotrophin-1 ELISA kits (Immuno-Biological Laboratories, Inc, IBL-America, Minneapolis). The intra-assay and inter-assay coefficient of variation were <10% and <10%, respectively. The sensitivity was <12 pg/ml. The detection range were 31.2–2000 pg/ml. For assay validation, another cardiotrophini-1 ELISA kit (ScienCell Research Laboratories, San Diego, CA, USA) was used in a subgroup of 80 age- and gender-matched subjects. Serum total cholesterol, triglycerides, and HDL levels were determined in the central laboratory of National Cheng Kung University Hospital with an autoanalyzer (Hitachi 747E; Hitachi, Tokyo, Japan). The estimated glomerular filtration rate (eGFR, ml/min/1.73 m2) was calculated using the Modification of Diet in Renal Disease (MDRD) equation.

## Statistics

SPSS software (version 17.0; SPSS, Chicago, IL) was used for statistical analysis. Continuous variables were expressed as means ± standard deviations or percentages. The cardiotrophin-1 concentration was expressed as the median (inter-quarter range), and log transformed before analysis. Study subjects were categorized into one of the following three groups: normal weight, overweight, and obese. The continuous variables among the three groups were compared using ANOVA or a Kruskal-Wallis test when the distribution of the variables was not normal. Chi-square tests were used to analyze the differences in categorical variables among groups. Multivariate linear regression analysis was conducted to identify the independent predictors of cardiotrophin-1 concentration. The independent variables included age, gender, BMI, FPG, insulin, SBP, total cholesterol, triglyceride, HDL, eGFR, ALT, smoking habit, and alcohol drinking habit. A *p* value less than 0.05 was considered statistically significant.

## Results

**Figure 1:**
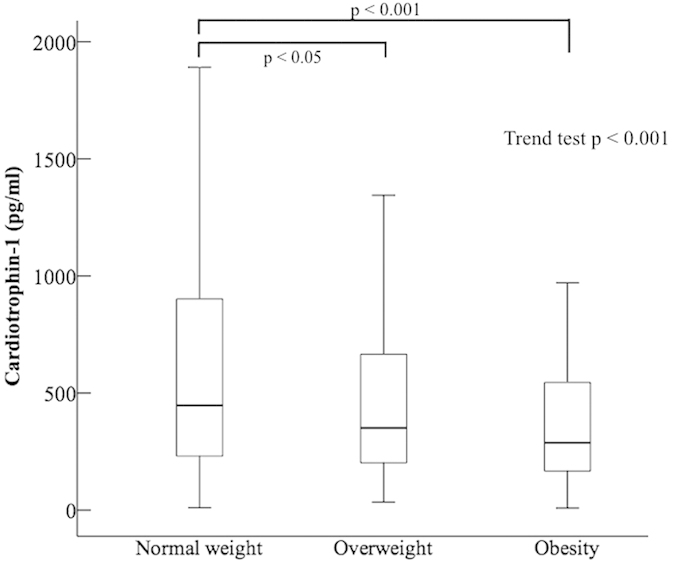
Comparisons of cardiotrophin-1 levels among subjects who were normal weight, overweight, and obese.

A total of 818 subjects who were normal weight (n = 522), overweight (n = 203), and obese (n = 93) were enrolled. The clinical characteristics of the study subjects are presented in Table 1. There were significant differences in age, BMI, SBP, DBP, FPG, PPG, insulin, HOMA-IR, triglyceride, HDL, ALT, AST levels, and the prevalence of female gender and hypertension among the three groups. The concentrations of cardiotrophin-1 were 447.9 (230.9, 913.9), 350.6 (201.1, 666.5), and 288.1 (162.3, 572.4) pg/ml in subjects who were normal weight, overweight, and obese, respectively (Fig. 1, ANOVA test *p* < 0.001, trend test *p* < 0.001). In the *post hoc* analysis, both subjects who were obese and overweight had significantly lower cardiotrophin-1 levels than those with normal weight.

The associations between cardiotrophin-1 and the various clinical characteristics were examined by the multivariate linear regression analyses. As shown in Table 2, BMI (beta = −50.036, 95% CI = −85.253 ~ −14.819, p < 0.05) and current smoking (beta = −387.122, 95% CI = −667.736 ~ −106.509, p < 0.01) were negatively related to cardiotrophin-1 levels after adjusting for age, gender, FPG, insulin, SBP, total cholesterol, HDL, triglyceride, eGFR, ALT, and alcohol drinking (model 1). In model 2, overweight (beta = −338.718, 95% CI = −552.786 ~ −124.651, p < 0.01), obesity (beta = −530.275, 95% CI = −832.967 ~ −227.583, p < 0.01), and current smoking (beta = −654.353, 95% CI = −654.353 ~ −100.397, p < 0.01) were negatively associated with cardiotrophin-1 after adjusting for age, gender, HOMA-IR, hypertension, total cholesterol, HDL, triglyceride, eGFR, ALT, and alcohol drinking

## Discussion

Our data revealed that obese and overweight individuals without diabetes had lower cardiotrophin1- levels than normal weight ones. Moreover, both obesity and being overweight were inversely associated with cardiotrophin-1. To the best of our knowledge, this is the first report to show that not only obesity, but also being overweight, are negatively associated with cardiotrophin-1 in non-diabetic subjects.

Natal *et al.* found that cardiotrophin-1 levels were higher in subjects with metabolic syndrome than the control group^10^, and those who met the hyperglycemia criteria (fasting glucose > 100 mg/dl) or the obesity criteria (BMI > 30 kg/m2) had higher cardiotrophin-1 levels than those who did not. Our previous study showed that subjects with impaired glucose tolerance and diabetes had significantly higher cardiotrophin-1 concentrations than those with normal glucose tolerance, and impaired glucose tolerance and diabetes were positively associated with cardiotrophin-1^11^. In addition, we found that obese, diabetic subjects had significantly lower cardiotrophin-1 levels than non-obese diabetic patients, and obesity was negatively associated with cardiotrophin-1^11^. However, cardiotrophin-1 levels were positively correlated with fasting plasma glucose, but not associated with BMI, in Natal *et al.* They might thus have overestimated the impact of obesity on cardiotrophin-1 concentrations, which was confounded by hyperglycemia. Moreover, Natal *et al.* did not run the multivariate regression analysis of cardiotrophin-1. Therefore, in the current work we excluded subjects with diabetes to minimize the positive effect of hyperglycemia on cardiotrophin-1, and the results showed that cardiotrophin-1 is inversely associated with BMI, or being overweight and obese, in non-diabetic individuals.

The expression of cardiotrophin-1 have been found in cardiomyocyte, skeletal muscle, liver, lung, pancreas, kidney, and adipose tissue in human^10^. Cardiotrophin-1 null mice develop obesity and recombinant cardiotrophin-1 treatment reduces body weight in *ob/ob* and in high-fat-fed obese mice^5^. However, the mechanisms underlying the role of cardiotrophin-1 in obesity are unclear. Obesity-induced cellular dysfunction activates a diverse range of signaling pathways, such as the activation of suppressor of cytokine signaling (SOCS) proteins, JNK, IKK, and ERK, and each pathway converges on and then inhibits the insulin signaling pathway and augments an inflammatory response within metabolic tissues^13^. *In vitro*, the gp130 cytokines bind to gp130 receptors and activate Jak/STAT signaling, while SOCS proteins are then activated to inhibit the signal transduction of the Jak/STAT pathway in a negative feedback loop^14^. Obesity may thus exert an inhibitory feedback pathway through the activation of SOCS to downregulate the expression of cardiotrophin-1. Moreover, several gp130 cytokines that use the leukemia inhibitory factor receptor (LIFR), including cardiotrophin-1, LIF, and human oncostatin M, can inhibit the subsequent signaling of other family members in adipocytes^15^. While the expression of LIF in obesity is unclear, oncostatin M expression is increased in adipose tissue from mice with diet-induced and genetic obesity and in obese humans^16^. These cytokines might exert inhibitory crosstalk on the expression of cardiotrophin-1.

There are several limitations to this work, as follows. First, since this study used a cross-sectional design, it did not allow for causal interpretation of the relationships found. Next, this population-based study aimed at finding epidemiological evidence of chronic diseases, and so adipose tissue was not obtained from the subjects. Therefore, the expression of other cytokines of the IL-6 family and the underlying cellular pathways could not be determined. Third, obesity is associated with chronic inflammation. Inflammatory cytokines, such as IL-6, were not measured. Whether these inflammatory cytokines played a role in the relationship between cardiotrophin-1 and obesity could not be determined. However, subjects with any acute or chronic inflammatory disease, as determined by a leukocyte count of more than 10,000/mm^3^ or clinical signs of infection were excluded from this study. Fourth, N-terminal pro-brain natriuretic peptide (NT-proBNP) was not measured. Subjects with heart failure might not have been excluded, despite those with past history or symptoms of heart failure, and left ventricular hypertrophy diagnosed by electrocardiogram were excluded. Finally, all the study subjects were Chinese, and the findings may not be generalizable to other ethnicities.

In conclusion, this study provided epidemiological evidence that non-diabetic subjects who were overweight or obesity had significantly lower cardiotrophin-1 concentrations than those with normal weight, and both obesity and being overweight were inversely associated with cardiotrophin-1 levels. Our data suggest that increased bodyweight might be related to the reduced cardiotrophin-1 status, and support the view that cardiotrophin-1 may be a promising therapy for obesity.

## Additional Information

**How to cite this article**: Hung, H.-C. *et al.* Cardiotrophin-1 is inversely associated with obesity in non-diabetic individuals. *Sci. Rep.* **5**, 17438; doi: 10.1038/srep17438 (2015).

## Figures and Tables

**Table 1:** Comparisons of clinical characteristics among subjects who were normal weight, overweight, and obese.

	Normal weight	Overweight	Obese	P
N (%)	522 (63.8)	203 (24.8)	93 (11.4)	—
Age (years)	45.1 ± 13.2	47.3 ± 12.8	49.4 ± 13.5	<0.01
Female (%)	63.2	40.9	45.2	<0.001
BMI (kg/m^2^)	21.5 ± 1.5	25.3 ± 0.8	29.2 ± 2.3	<0.001
SBP (mmHg)	111.7 ± 16.6	120.3 ± 14.9	127.4 ± 16.5	<0.001
DBP (mmHg)	66.7 ± 9.5	72.1 ± 9.6	74.3 ± 9.3	<0.001
Hypertension (%)	7.9	13.3	17.2	<0.01
FPG (mmol/l)	5.3 ± 0.5	5.5 ± 0.7	5.5 ± 0.6	<0.001
PPG (mmol/l)	6.5 ± 1.5	7.0 ± 1.5	7.2 ± 1.7	<0.001
Insulin (mU/l)	3.69 ± 2.42	5.18 ± 2.72	7.79 ± 5.24	<0.001
HOMA-IR	0.88 ± 0.59	1.28 ± 0.73	1.94 ± 1.44	<0.001
Total cholesterol (mmol/l)	5.1 ± 1.1	5.2 ± 1.0	5.3 ± 1.1	NS
Triglyceride (mmol/l)*	1.1 ± 0.8	1.5 ± 0.8	1.8 ± 1.1	<0.001
HDL cholesterol (mmol/l)	1.5 ± 0.4	1.2 ± 0.4	1.2 ± 0.3	<0.001
eGFR (ml/min/1.73 m^2^)	88.1 ± 15.8	85.3 ± 15.5	87.0 ± 15.6	NS
AST (U/L)	21.9 ± 12.6	24.9 ± 14.1	27.5 ± 18.3	<0.001
ALT (U/L)	23.3 ± 23.7	30.1 ± 24.0	33.0 ± 19.6	<0.001
Current smoker (%)	12.8	17.7	18.3	NS
Current alcohol drinker (%)	9.4	12.8	10.8	NS

Data are expressed as means ± SD or %.

*Kruskal-Wallis test.

Abbreviations: NS, not significant; BMI, body mass index; SBP, systolic blood pressure; DBP, diastolic blood pressure; FPG, fasting plasma glucose; PPG, Post-load 2-h plasma glucose; HOMA-IR, homeostatic model assessment-insulin resistance; eGFR, estimated glomerular filtration rate; AST, aspartate aminotransferase; ALT, alanine aminotransferase.

**Table 2:** Multivariate linear regression analyses of cardiotrophin-1 and clinical variables.

	Model 1		Model 2	
	β (95% CI)	p value	β (95% CI)	p value
Age (years)	0.486 (−8.024 ~ 8.996)	NS	−0.134 (−7.985 ~ 7.717)	NS
Gender, male vs. female	1.440 (−213.559 ~ 216.438)	NS	−10.896 (−221.746 ~ 199.954)	NS
BMI (kg/m^2^)	−50.036 (−85.253 ~ −14.819)	<0.05	—	
Overweight, yes vs. no	—		−338.718 (−552.786 ~ −124.651)	<0.01
Obese, yes vs. no	—		−530.275 (−832.967 ~ −227.583)	<0.01
FPG (mmol/l)	−21.944 (−191.530 ~ 147.642)	NS	—	
Insulin (mU/l)	−7.184 (−38.237 ~ 23.870)	NS	—	
HOMA-IR	—		−33.312 (−147.699~81.074)	NS
SBP (mmHg)	−2.811 (−9.271 ~ 3.650)	NS	—	
Hypertension, yes vs. no	—		−171.381 (−479.792 ~ 137.031)	NS
Total cholesterol (mmol/l)	34.136 (−65.625 ~ 133.897)	NS	28.091 (−70.540 ~ 126.721)	NS
HDL cholesterol (mmol/l)	−77.691 (−383.544 ~ 228.162)	NS	−96.572 (−399.041 ~ 205.898)	NS
Triglyceride (mmol/l)	75.598 (−407.435 ~ 558.631)	NS	94.055 (−385.627 ~ 573.737)	NS
eGFR (ml/min/1.73 m^2^)	2.544 (−3.817 ~ 8.904)	NS	2.125 (−4.190 ~ 8.440)	NS
ALT (U/L)	2.784 (−0.903 ~ 6.471)	NS	2.887 (−0.779 ~ 6.553)	NS
Current alcohol drinker, yes vs. no	215.051 (−98.628 ~ 528.730)	NS	199.991 (−112.732 ~ 512.715)	NS
Current smoking, yes vs. no	−387.122 (−667.736 ~ −106.509)	<0.01	−377.375 (−654.353 ~ −100.397)	<0.01

Dependent variable: cardiotrophin-1.

Abbreviations: NS, not significant; BMI, body mass index; SBP, systolic blood pressure; FPG, fasting plasma glucose; HOMA-IR, homeostatic model assessment-insulin resistance; eGFR, estimated glomerular filtration rate; ALT, alanine aminotransferase.
